# Intestinal microsporidiosis in Iran: infection in immune-compromised and immunocompetent patients 

**DOI:** 10.29252/cmm.3.1.30

**Published:** 2017-03

**Authors:** M Nooshadokht, I Sharifi, MA Mohammadi, M Pirestani, A Afgar, A Mahootchi, S Salari

**Affiliations:** 1 Department of Medical Parasitology and Mycology, School of Medicine, Kerman University of Medical Sciences, Kerman, Iran; 2 Leishmaniasis Research Center, Kerman University of Medical Sciences, Kerman, Iran; 3 Research Center for Hydatid Disease in Iran, Kerman University of Medical Sciences, Kerman, Iran; 4 Department of Parasitology and Entomology, School of Medicine, Tarbiat Modares University, Tehran, Iran; 5 Regional Knowledge Hub and WHO Collaborating Centre for HIV Surveillance, Kerman University of Medical Sciences, Kerman, Iran; 6 Department of Pathobiology, School of Veterinary Medicine, Shahid Bahonar University of Kerman, Kerman, Iran

**Keywords:** Immunocompetent individuals, Immunocompromised patients, Iran, *Microsporidia*

## Abstract

**Background and Purpose:**

Gastroenteritis and the clinical profile caused by *Microsporidia*, an opportunistic pathogen, may be severe in immunocompromised individuals, especially in AIDS patients. Conventionally, it is necessary to detect the small infective spores in stained smears. However, due to the limitations of the microscopy-based methods, several DNA-based methods such as polymerase chain reaction (PCR) have recently been developed to enhance diagnosis sensitivity. Therefore, we sought to evaluate the rate of infection in immunocompromised patients as compared with immunocompetent patients in Kerman, Iran.

**Materials and Methods:**

We collected stool samples of 199 human subjects (116 males and 83 females), aged 1 to 69 years old. They were divided into immunocompromised (i.e., AIDS [n=72] and cancer-positive patients [n=59]) and immunocompetent (n=68) groups. We comparatively examined the fecal materials using the microscopy and PCR methods.

**Results:**

The overall prevalence rate of *Microsporidia* infection was 10.05% (20/199). *Entrocytozoon bieneusi *was the only species within the *Microsporidia *genus that was identified in 14.5% (19/131) of the immunocompromised patients and 1.47% (1/68) of the immunocompetent individuals.

**Conclusion:**

Considering the higher prevalence rate of microsporidiosis in patients with immunodeficiency (10.03%), we suggest performing sensitive and specific techniques such as PCR for the detection of these parasites in immunocompromised patients.

## Introduction

*Microsporidia* is an intracellular eukaryotic microorganism that infects numerous animal and human cells [1]. Consistent with the results of recent phylogenetic and genetic analyses, the hypothesis that *Microsporidia* may be real fungi has been supported. It seems that this species has a very special evolutionary relationship with zygomycetes [2, 3].

Two species of *Microsporidia*, including *Enterocytozoon bieneusi* and *Encephalitozoon intestinalis,* are the most common etiological agents of intestinal infection in humans [4]. Immunocom-promised, especially AIDS, patients with CD4^+ ^T cell count of less than 100 /µl are at high risk for this type of infection. In this group of patients, the infection can be life-threatening [4, 5].

The course of infection and its response to treatment is generally different in immunocompetent individuals in comparison with AIDS patients. Besides, the former group is able to control the infectioneven without treatment [6, 7]. Other than AIDS, immunodeficiency disorders such as neoplastic diseases are the risk factors for the development of severe forms of microsporidiosis. 

There is a necessity for the use of more sensitive diagnostic tests than microscopy because microscopic examination has some limitations in detecting very small *Microsporidia *spores [8].

In the past decade, the staining and molecular-based methods for identifying the microorganism have improved exponentially [6, 9]. In the present study, the prevalence of intestinal microsporidiosis in immune-compromised and immunocompetent patients was examined using different protocols, in Kerman, Iran.

## Materials and Methods

This cross-sectional study was performed at Kerman University of Medical Sciences, Kerman, Iran. The participants were classified into three groups of cancer patients (n=59), HIV-positive patients (n=72) visiting hospitals for follow-up examination and treatment, and immunocompetent patients with diarrhea as the control group (n=68). Three stool specimens were collected from each of the 199 participants. 

The study was approved by the Ethics Committee (IR.KMU.REC.1394.325) of Kerman University of Medical Sciences. 

***Stool specimens and processing***

Stool samples were immediately transported to the parasitology laboratory and divided into several portions according to the type of examination.

***Ryan Blue modified trichrome staining method***

First, two smears were prepared from 10 µl of fresh or concentrated stool samples on glass slides followed by spreading an area measuring about 25×45 mm. The air-dried smears were fixed with absolute methanol for 5 min, placed in the trichrome stain for 90 min, and then rinsed in alcohol-acid (1% glacial acetic acid in 96% ethanol) for 10 s. The samples were rinsed several times with 95% ethanol followed by dehydration with 95% and absolute ethanol for 5 and 10 min, respectively. The smears were placed in xylene for 10 min, and finally, they were mounted and covered with coverslips in the last step [10]. 

***Genomic DNA extraction***

Microsporidian spore walls were mechanically disrupted using glass beads (0.45-0.52 mm in diameter), and lysed by vortexing and freezing and thawing. After addition of 25 µl of proteinase K, the suspension was incubated at 56ºC for 4-6 h followed by using QIAamp DNA stool mini kit (Qiagen Company, Germany) for DNA extraction as described by the manufacturer’s instructions. The concentration and purity of the extracted DNA were measured with optical density (OD) ratio at 260/280 nm by using a NanoDrop spectrophotometer (Thermo Fisher Scientific). All the extracted DNAs were stored at -20ºC. 

***PCR for the detection of Microsporidia***

Four primers were used for *E. bieneusi* and *E. intestinalis* amplification as described by Lejeune et al. and Menotti et al., respectively. Amplification of the small subunit rRNA gene was performed using the forward primer, Eb1, (5' CGACAGCCTGTGTGTGAGAATAC 3') and the species-specific reverse, Eb5, (5' CAACGAATGACTTGACCCTGGTAA 3') for the diagnosis of *E. bieneusi* [11]. The primer sets FEI1 (5' GCAAGGGAGGAATGGAACAGAACAG 3') and REI1 (5' CACGTTCAGAAGCCCATTACACAGC 3') amplified a 127 bp fragment specific for *E. intestinalis* [12]. The PCR reaction mixture consisted of 10 µl of 2x Master Mix RED (Ampliqon, Copenhagen, Denmark), which consisted of 1.5 mM MgCl_2_, 10 pmol of each primer, and 20 ng of DNA template in a final volume of 20 µl. Distilled water was used as the negative control.

DNA was amplified using a PCR machine (Analytik, Jena, Germany) under the following condition: 10 min at 94 ºC, followed by 35 cycles of 30s at 94 ºC, 20s at 61.5ºC, 30s at 72 ºC, and a final extension at 72ºC for 10 min. The PCR products were separated on 2% agarose (Takara Shuzo, Kyoto, Japan). 

The gels were stained with ethidium bromide(0.1 mg/ml) and visualized using UV transilluminator (UVItec, Cambridge, UK). The bands of 127 bp and 180 bp showed infection with* E. bieneusi* and *E. intestinalis*, respectively. The PCR products were separated on 2% agarose gel (Takara Shuzo, Kyoto, Japan) in 0.5x Trisborate-EDTA buffer, stained with ethidium bromide (0.1 mg/ml), and visualized under UV light (UVItec, Cambridge, UK). 

***CD4*** ^+^ ***T cell count***

Peripheral blood of HIV-positive patients was collected into EDTA tubes and 20 µl of it was stained with a fluorescent anti-CD4 monoclonal antibody. The CD4^+^ T cell whole count was performed by a flow cytometer.

***Data analysis***

To analyze the data, Chi-square test, Pearson correlation coefficient, Fisher's exact test, and logistic regression were run in SPSS, version 21.

## Results

**Figure 1:**
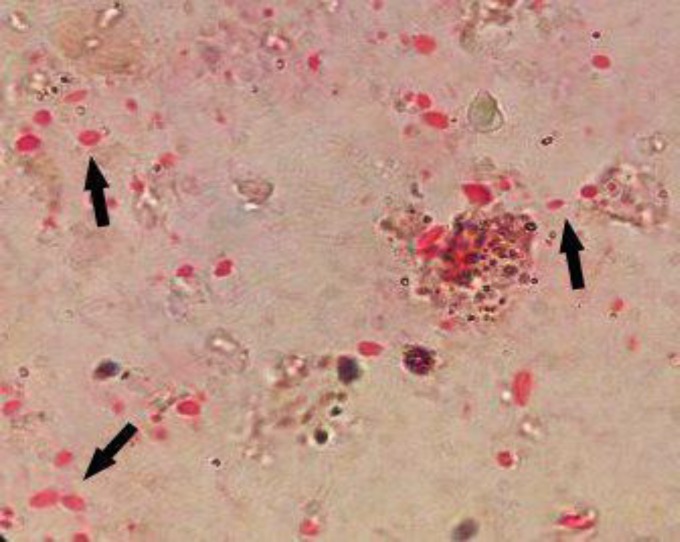
Modified trichrome staining of* Enterocytozoon bieneusi *spores isolated from the stool samples; 1000x magnification

**Figure 2:**
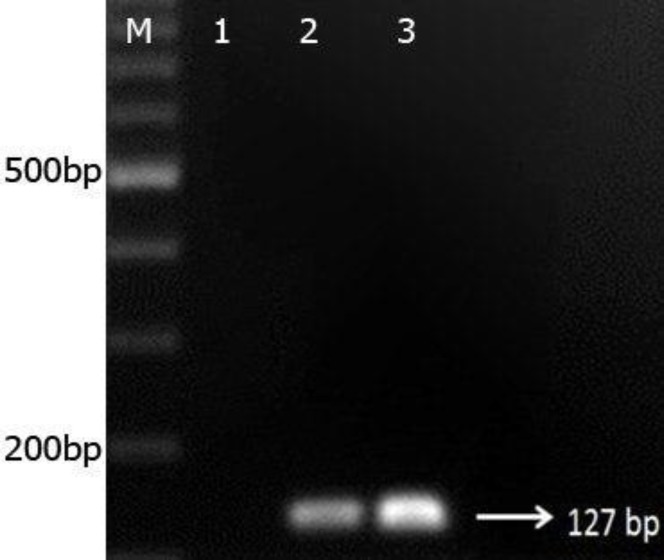
Agarose gel electrophoresis (2%) of random PCR products (127 bp). Lane 1: negative control; lane 2: positive control; lane 3: PCR products of positive samples; lane M: 100-bp DNA ladder

The overall frequency of *Microsporidia* spp. infection among the participants as detected by microscopy (modified trichrome stain; Figure 1) and DNA-based PCR amplification was 10.5% (20/199). Microscopy and PCR yielded identical results (PCR was selected as the gold standard test), and all the DNAs extracted from positive isolates detected using Ryan trichorome stain were amplified well. *E. bieneusi* was the only species in *Microsporidia* genus that was identified. In this study, the use of specific primers on the 18S rRNA locus of *E. bieneusi* resulted in 127–bp PCR products (Figure 2); whereas no band of 180 bp was observed using the specific primers of *E. intestinalis*. Using the specific primers and the PCR method, 1/68 (1.47%), 10/72 (13.88%), and 9/59 (15.28%) of the immunocompetent controls, HIV-positive, and cancer-positive patients were infected with *E. bieneusi*, respectively. 

The results showed that the prevalence of *E. bieneusi* was significantly different in the immunodeficient and immunocompetent patients based on their immune state and it was higher in the former group )*P=0.002*, CI 95%: 1.49-86.85; OR=11.37). Although there was no significant difference in the prevalence of *Microsporidia* infection among patients with different cancer types (*P>0.05*), it was more frequent among patients with leukemia (25%) followed by gastrointestinal cancer (22.2%). In addition, in HIV-positive patients, no significant difference was noted in terms of CD4^+^ cells count level and microsporidiosis frequency (*P>0.05*). Nonetheless, half of the HIV-positive cases (5/10) who were infected with *Microsporidia *had TCD4^+^ cell count < 200 µl.

The highest and lowest prevalence rates of *Microsporidia* infection were respectively observed in the 40-49 (7, 13.5% ) and 1-9 (1, 9.1% ) and 20-29 (1, 3.8%) age groups. However, no significant differences were found among different age groups with respect to *Microsporidia* infection rate (*P<0.05*; Table 1).

Overall, the risk of *Microsporidia* morbidity among patients with diarrhea was 5.99 times higher than those without diarrhea and it was 5.18 times greater in cases with abdominal pain than the asymptomatic individuals (*P=0.002* and *P=0.007*, respectively). In addition, the rate of recurrence of *E. bieneusi* infection was compared among the three groups based on their clinical symptoms, and it was found that the HIV-positive group was significantly different in this regard (*P=0.001*; Table 2).

The results showed that out of the 199 precipitants, 116 and 83 individuals were male and female, respectively. Out of the116 males, *E. bieneusi* was detected in 13 (11.2%) cases, while in the 83 females, 7 (8.4%) were infected with *E. bieneusi*. The results indicated no significant differences between males and females in *Microsporidia* infection (*P>0.05*, CI 95%: 0.522-3.6; Table 3). 

**Table 1:** The frequency of *E.bieneusi* in the participants based on their immune status and age

***P-value***	**SEM**	**SD**	**Mean age (Year)**	**N**	***E.bieneusi***	**Subjects **
0.287	2.10	17.16	33.57	67	Negative	Immunocompetent
	-	-	15	1	Positive	
0.326	2.47	20.30	41.06	50	Negative	Cancer-positive
	8.16	24.47	33.56	9	Positive	
0.84	1.23	9.72	39.838	62	Negative	HIV/AIDS-positive
	2.66	8.40	40.50	10	Positive	

SD: Standard deviation; SEM: Standard Error of Mean; Significant difference at *P*<0.05

**Table 2:** The frequency of *E.bieneusi* in the participants based on their clinical symptoms

***P*** **-** ***value***	**95% CI for OR**	**OR**	***E.bieneusi***		**Total**	**Clinical symptoms**	
			**Positive (%)**	**Negative (%)**			
0.002*	1.70 21.17))	1	3(3.2%)	92(96.8%)	95(100%)	Negative	Diarrhea
		5.99	17(16.3%)	87(83.7%)	104(100%)	Positive	
			20(10.1%)	179(89.9%)	199(100%)	Total	
0.002*	1.67 16.11))	1	4(3.8%)	101(96.2%)	105(100%)	Negative	Abdominal pain
		5.18	16(17%)	78(83%)	94(100%)	Positive	
			20(10.1%)	179(89.9%)	199(100%)	Total	
0.509	0.539 3.47))	1	11(8.9%)	112(91.1%)	123(100%)	Negative	Vomiting
		1.37	9(11.8%)	67(88.2%)	76(100%)	Positive	
			20(10.1%)	179(89.9%)	199(100%)	Total	

* Significant difference at *P*<0.05

**Table 3:** The frequency of *E.bieneusi* in the participants based on gender

***P-value***	**Total**	***E.bieneusi***		**Sex**	**Subjects **
		**Positive** **N (%)**	**Negative** **N (%)**		
0.471	36(100)	0(0)	36(100)	Female	Immunocompetent
	32(100)	1(3.1)	31(96.9)	Male	
	68(100)	1(1.5)	67(98.5)	Total	
0.999	21(100)	3(14.3)	18(85.7)	Female	Cancer-positive
	38(100)	6(15.8)	32(84.2)	Male	
	59(100)	9(15.3)	50(84.7)	Total	
0.998	26(100)	4(15.4)	22(84.6)	Female	HIV/AIDS-positive
	46(100)	6(13)	40(87)	Male	
	72(100)	10(13.9)	62(86.1)	Total	

Significant difference at *P*<0.05

## Discussion

*Microsporidia* are one of the known causes of opportunistic infections in humans, especially in immunodeficient individuals. The prevalence of this infection ranges between 0% and 50% worldwide [13]. Parallel to the growing number of immunodeficient patients and the recognition of the HIV virus, the prevalence of infectious diseases has been on an uprising trend in the past few decades [14, 15]. Cancer-positive patients, transplant recipients, and especially those with AIDS, are the most susceptible groups to microbial infections. Moreover, insufficient immune system response can affect parasite clearance in these subjects [15]. A limited number of studies has been conducted on the incidence of intestinal microsporidiosis in AIDS and cancer patients, as well as transplant recipients [16, 17].

In this study, the overall prevalence of *Microsporidia* spp. infection was 10.05%. It was detected in 14.5% of the immunocompromised patients (13.88% and 15.28% in the HIV-positive and cancer-positive subjects, respectively) and in 1.47% of the immunocompetent cases with diarrhea. It is worth mentioning that in HIV-positive patients, access to care programs may reduce the infection rate. In our study, although we found a relatively high prevalence of 13.88% in HIV-positive patients, they all had received the anti-retroviral drug. However, this rate was lower than the prevalence rates reported in some former studies. For example, Mirjalali et al. detected *E. bieneusi* in 30.86% of the Iranian HIV-positive subjects [6]. However, Agholi et al. found *E. bieneusi *in 2.24% of HIV-positive patients in Shiraz, Iran [18].

*E. bieneusi* was the single species that was recognized in our study. Several studies have also reported that* E. bieneusi* was the most common agent of gastroenteritis [6, 19]. In Peru, Bern et al. investigated intestinal *Microsporidia* infection rate in patients with AIDS. In their study*, E. bieneusi* was also identified as the only *Microsporidia* [20]. Mirjalali et al. detected *E. bieneusi* and *E. intestinalis* and demonstrated that*E. bieneusi* was the most important cause of gastroenteritis. Kazemi et al. performed a study to identify the prevalence of microsporidiosis in the southwest of Iran. They reported both *E. bieneusi* and *E. intestinalis* in the samples [19]. In contrast, in a study in India, *E. intestinalis* was the most common cause of microsporidiosis in patients with diarrhea [21].

The results of our study indicated that the risk of *Microsporidia *morbidity among the patients with diarrhea was 5.99 times higher than those without this symptom and it was 5.18 times greater in those with abdominal pain than the asymptomatic individuals. Moreover, there was a significant difference between immunocompromised patients and healthy individuals in terms of prevalence of *Microsporidia*. The results of this study were consistent with those of some other studies. Several studies confirmed the association between microsporidiosis or other intestinal parasitic infections and gastrointestinal diseases in immune-deficient patients [22, 23]. Moreover, the results of Samie et al. revealed that *Microsporodia* infection rate was higher in patients with diarrhea than others [24], which is in line with the current findings. Coyle et al. identified an association between microsporidiosis and diarrhea. They concluded that *Microsporidia* contaminated 44% of HIV-positive patients with diarrhea [25]. Similarly, studies were performed in Africa to examine the prevalence and clinical manifestations of intestinal microsporidiosis in AIDS patients in comparison to healthy subjects [26, 27]. They reported that* E.* *bieneusi* was the most common species in symptomatic patients. Conversely, Lobo et al. did not report any significant differences among immunocompromised subjects in *Microsporidia* infection rate based on clinical symptoms [28]. Further, in our study, the incidence of microsporidiosis in HIV-positive group did not significantly increase with declining CD4^+^ T cell count level.

The reports on the incidence of microsporidiosis in cancer-positive patients are insufficient [28]. It seems that those taking immunosuppressive drugs are more susceptible to infection presumably because their immune response is affected by these drugs [19]. We found that the prevalence of *E.* *bieneusi* was significantly different based on patients' immune state. The incidence of microsporidiosis in immunocom-promised patients was significantly higher than immunocompetent individuals. In our study, the prevalence of *E.* *bieneusi* in cancer-positive cases was 15.28%. However, there was no significant difference in the frequency of microsporidiosis among the subjects with different cancer types. Nonetheless, the incidence of the disease was higher in patients with leukemia and gastrointestinal cancer than others. Angela et al. also detected *Microsporida* infection in 68/217 (31.33%) cancer-positive patients and only 5/144 (3.47%) healthy cases. The difference between the two groups was significant [29]. Kezemi et al. presented that cancer-positive patients were more at risk for microsporidiosis. They detected *Microsporidia* in 18% of these cases [19]. In our study, there was no significant difference in microsporidiosis rate between males and females. This finding was consistent with the results of studies performed in Malaysia and India. The authors also reported that even though the number of women who were positive for microsporidiosis was higher than men, the difference was not statistically significant [21]. On the contrary, in a study carried out by Tabatabaei in Iran, the infection rate was significantly higher in men than in women [30].

In addition, it should be mentioned that various factors such as the type of diagnostic method, the distribution of different individuals in the studied groups, and the diversity of species or genotypes of parasites may affect the results. One of the important factors is the type of laboratory methods used to identify the microorganism. There are several tests available to identify intestinal parasitic infections. Microscopy is the conventional diagnostic tool in parasitology laboratories; however, it has low sensitivity and specificity due to the following reasons. First, it is highly dependent on the operator's expertise. Diagnosis of small microorganisms or their forms such as microsporidian spores is very difficult using microscopic examination. In addition, degenerated or atypical spores may not be detected easily in the specimens. These factors compromise the sensitivity of the microscopy method. Second, the detection of *Microsporidia* species based on shape is usually difficult or impossible in direct examination, hence small spores must be observed in stained smears [31]. Third, the conventional examinations are not often used for the discrimination of these parasites; therefore, requesting specific diagnostic tests is necessary [32]. 

In recent years, researchers have focused on improving the diagnostic methods at the species/genotype level [33-35]. Various diagnostic assays, including PCR, have become available for the identification of parasites or fungi. Conventional PCR has been compared to microscopic examination for the identification of several microorganisms in clinical specimens [36]. This study compared conventional microscopy and PCR detection methods for the detection of *Microsporidia*. PCR is considered the gold standard as it has the highest sensitivity and specificity in identifying parasites. In this study, the results of microscopy and PCR were identical. However, DNA-based techniques do not require integration microorganisms; thus, the assays enable monitoring any parasite DNA load in feces. Consequently, molecular-based protocols can be a good alternative to traditional methods in most laboratories, especially in a large scale. Nevertheless, there are some problems with using PCR-based methods in Iran. First, the method are not readily available, and second, the costs of performing these tests are higher than the other routine parasitology tests. In addition, PCR assay is unable to discriminate between the present and chronic forms of the disease. 

## Conclusion

As of yet, the prevalence rate of microsporidiosis has not been fully recognized in cancer-positive patients in Iran. However, there are a few studies on the prevalence rate of this infection among AIDS patients. In this study, we evaluated the prevalence of an important opportunistic parasite/fungal species, that is *Microsporidia*, in both immunocompromised (AIDS- and cancer-positive patients) and immunocompetent individuals. Since understanding the actual status and epidemiology of a parasite is important for decision-making, planning, and control strategies, we suggest the routine implementation of at least one of the procedures of PCR, ELISA, and/or staining method for the detection of *Microsporidia* spp. in immunocom-promised patients in the reference laboratories, academic centers, or children's hospitals. Overall, we conclude that modified trichorome staining methodis the suitable technique for the detection of *Microsporidia* spp. in most laboratories. 
